# Utilization of Contrast-Enhanced Ultrasound in Diagnosis of Focal Liver Lesions

**DOI:** 10.1155/2024/3879328

**Published:** 2024-10-24

**Authors:** Fady Salama, Nimish Thakral, Christina Delacruz Leyson, Venkata Konjeti, Karim Benrajab, Gregory Hawk, Harrison Fouch, Roberto Gedaly, Aman Khurana

**Affiliations:** ^1^Department of Internal Medicine-Division of Digestive Diseases and Nutrition, University of Kentucky, Lexington, Kentucky 40536, USA; ^2^Dr. Bing Zhang Department of Statistics, University of Kentucky, Lexington, Kentucky 40536, USA; ^3^College of Medicine, University of Kentucky, Lexington, Kentucky 40536, USA; ^4^Department of Surgery-Division of Hepatobiliary and Transplant Surgery, University of Kentucky, Lexington, Kentucky 40536, USA; ^5^Department of Radiology-Division of Abdominal Radiology, University of California San Diego, San Diego, California 92109, USA

**Keywords:** benign liver tumor, contrast-enhanced US, HCC, indeterminate liver lesion

## Abstract

**Background and aims**: Focal liver lesions (FLL) are one of the most common indications for hepatology and hepatobiliary surgery consultation. In this retrospective study, we aim to assess if contrast-enhanced ultrasound (CEUS) can address diagnostic dilemmas in the evaluation of indeterminate liver lesions by identifying characteristics of indeterminate FLL on CEUS and correlating these with cross-sectional imaging and pathology findings.

**Methods**: We retrospectively reviewed all patients who underwent CEUS evaluation for liver lesions over a 28-month period (Oct 2020 to Jan 2023) at the University of Kentucky. To assess the relationship between CEUS results and the corresponding CT, MRI, and/or pathologic findings, the McNemar–Bowker tests were performed.

**Results**: Twenty-nine patients were included (after two exclusions from a total *n* of 31). Mean age was 54 years, 62% were female, and 48% had underlying cirrhosis. Of the 29 patients with initial cross-sectional imaging, the initial results showed malignancy or likely malignant lesion in 6 patients and benign or likely benign lesion in 6 patients. The remaining 17 patients had inconclusive/indeterminate results. CEUS clarified an “indeterminate” CT/MRI result 15 times out of 17 (88.2%), moving the diagnosis to “benign” 11 times while suggesting “malignant” only four times. When aggregating indeterminate cross-sectional results with either benign or malignant categories suggested by CEUS, CEUS never reversed a benign CT/MRI diagnosis but often reversed a malignant CT/MRI diagnosis.

**Conclusion**: CEUS provided a definitive diagnosis of indeterminate liver lesions in approximately 90% of patients and avoided the need for biopsy in most patients. In cases where the liver lesions were biopsied, CEUS accurately distinguished malignant versus benign lesions as confirmed by biopsy findings. CEUS, therefore, has the potential to provide a precise diagnosis for the majority of indeterminate lesions.

## 1. Introduction

Focal liver lesions (FLL) frequently lead to hepatology and hepatobiliary surgery consultations, making them a prevalent clinical concern. These lesions are frequently discovered incidentally during abdominal imaging for unrelated issues or as part of surveillance in patients with cirrhosis. The incidence of FLL has increased remarkably with some studies reporting that incidental lesions are diagnosed in up to 33% of radiological studies [1]. Remarkably, necropsy studies have estimated a prevalence of up to 50% in the general population [2]. These FLLs often require multiple sequential diagnostic imaging modalities to obtain an accurate diagnosis. Traditionally, the assessment of FLLs has relied on cross-sectional imaging techniques such as computed tomography (CT) and magnetic resonance imaging (MRI), which offer high levels of accuracy. However, because of cost and accessibility, abdominal ultrasound (US) continues to be the first-line imaging modality of choice. In certain cases, abdominal ultrasound may not provide complete characterization of the FLL, necessitating the essential use of CT or MRI. However, CT/MRI can be limited by several factors including radiation exposure, the presence of ferromagnetic prosthesis/implants, contrast allergies, claustrophobia, and altered renal function.

Contrast-enhanced ultrasound (CEUS) is a noninvasive rather inexpensive method to evaluate FLL. The currently used contrast agents (second generation, Lumason, Bracco Industries) are gas-filled microbubbles (sulfur hexafluoride) stabilized by an outer shell made of albumin, surfactant, or phospholipids with excellent safety profile. The bubbles are less than 10 *μ*m to enhance microvasculature as they can freely circulate in the capillary beds while remaining strictly intravascular [3, 4]. These microbubbles are eliminated by gaseous diffusion in the lungs and/or liver metabolism [5]. As such, the evaluation of liver and renal function prior to contrast administration with this procedure is not absolutely warranted. Minimal side effects such as mild eye/skin irritation, headache, itching, and metallic taste have been reported and in rare cases, severe allergic reactions may occur [4, 6]. For that reason, Lumason is contraindicated in patients with a history of hypersensitivity reactions to sulfur hexafluoride lipid microsphere components or to any of the inactive ingredients in Lumason.

Given the safety characteristics of the CEUS technique, it has recently been considered in the evaluation of FLL of patients with abnormal renal function due to acute kidney injury or chronic kidney disease, and those with a prior history of allergy to iodine-based or gadolinium-based contrast agents. It, therefore, represents an increasingly relevant diagnostic method in everyday clinical practice. Our aim was to assess the role of CEUS in the evaluation of indeterminate FLL and the correlation between imaging and pathologic findings at a single tertiary care academic medical center.

## 2. Methods

After institutional review board approval (#67188), we searched our electronic medical record (EMR) database to include every consecutive patient who underwent CEUS for further characterization of benign and malignant hepatic lesions over a 28-month period (Oct 2020–Nov 2023) at the University of Kentucky (UK). In all cases, Lumason was used as a contrast agent for CEUS following a prior CT or MRI imaging study. After locating the lesion on conventional grayscale US images, two intravenous contrast injections of 2.2 mL each were performed with Lumason in two different US planes (transverse and sagittal) to better evaluate the enhancement and washout characteristics given 2D imaging limitations of standard US probes. This was also followed by a 5-10 mL saline flush for each injection to ensure microbubbles were not trapped in the intravenous cannula tubing. Briefly, cine images were obtained for the first minute post contrast injection and thereafter brief 5–10 s cine images were obtained at every minute time point for a total of 6 min postcontrast injection. Images were evaluated by an expert abdominal radiologist and reviewed at the institution's hepatobiliary tumor board.

Twenty-two patients had MRI, and seven patients had CT imaging prior to CEUS evaluation. Two patients did not complete cross-sectional imaging secondary due to severe contrast allergy.

### 2.1. Statistical Analysis

Descriptive statistics were calculated for patient demographics and lesion size. CEUS and CT/MRI findings were categorized as “malignant,” “benign,” or “inconclusive.” To assess the relationship between CEUS results and the corresponding CT, MRI, and/or pathologic findings, the McNemar–Bowker tests were performed. Additionally, exact binomial tests were used to compare agreement rates between methods, utilizing a null value of 0.5 (50% agreement). To investigate the robustness of our findings with respect to the subjective nature of scan readings, inconclusive results were handled in three different ways: as a distinct (third) category, aggregated with “malignant” readings, and aggregated with “benign” readings. Across all analyses, a *p* value of less than 0.05 was considered significant. All analyses were completed in R 4.1.2 (R Foundation for Statistical Computing; Vienna, Austria).

## 3. Results

A total of 31 patients were identified who underwent CEUS for evaluation of indeterminate FLL in the 28-month period (Oct 2022 to Jan 2023) at our institution. Two patients were excluded from the analysis due to the absence of either a biopsy or cross-sectional imaging. Out of 29 patients included, the mean age was 54 ± 15.4 years, 18 were female, and 14 had underlying cirrhosis.

The underlying chronic disease of the liver was distributed as follows: 10 patients had nonalcoholic fatty liver disease (NAFLD), 4 patients had alcohol-related liver disease, 4 patients with chronic HCV infection, 1 patient with chronic HBV infection, 1 patient was postliver transplantation, and 9 patients with no history of liver disease (Table 1).

Twenty-two patients had MRI and seven patients had CT imaging prior to CEUS evaluation. Two patients did not complete cross-sectional imaging secondary due to severe contrast allergy.

**Figure 1:**
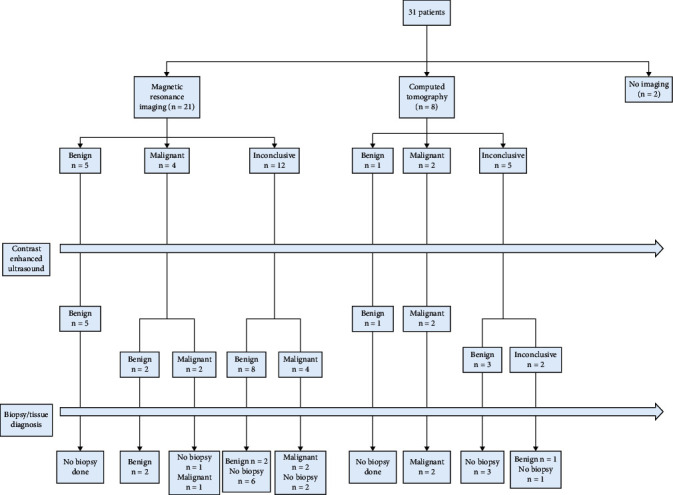
Results of cross-sectional CT/MRI correlated to CEUS and tissue diagnoses.

Of the 29 patients with initial cross-sectional imaging, the initial results showed malignancy/likely malignant lesion in 6 patients and benign/likely benign lesion in 6 patients. The remaining 17 patients had inconclusive/indeterminate results (Figure 1).

Overall, the CEUS results matched the initial cross-sectional imaging results in 12 of 29 cases (41.4%, value = 0.867). Of the 6 patients whose initial cross-sectional imaging results suggested possible malignancy, CEUS detected malignancy in just 67% of the lesions (4 patients, 3 of them with confirmed on biopsy). The other 2 patients showed benign features on CEUS and the biopsy confirmed no malignancy, confirming CEUS features over cross-sectional imaging.

All six lesions that were initially characterized as benign on cross-sectional imaging were also identified as nonmalignant using CEUS. None of those 6 patients underwent liver lesion biopsy.

A total of 10 patients out of 29 underwent liver lesion biopsy based on CEUS results. In 9 out of 10 patients in whom a biopsy was performed, the histopathological findings were consistent with CEUS results (90%, value = 0.011). One patient with inconclusive CT and CEUS results had benign pathology on biopsy.

Seventeen lesions were initially characterized as indeterminate lesions on cross-sectional imaging. CEUS clarified an “indeterminate” CT/MRI result 15 times out of 17 (88.2%), moving the diagnosis to “benign” 11 times while suggesting “malignant” only 4 times. An “indeterminate” result is somewhere between a definitive “benign” diagnosis and a definitive “malignant” diagnosis. This marked reduction in “indeterminate” cases in the CEUS results was the driving force behind the statistically significant asymmetry in the relationship between the paired CEUS and CT/MRI results (*p* value = 0.0007). It also explains the poor agreement between the two methods.

Interestingly, when “indeterminate” results were combined with “benign” results, this significant asymmetry between the two methods vanished (*p* − value = 0.683) and we observed an agreement increase with 23 out of 29 patients (79.3%, *p* − value = 0.001). However, when “indeterminate” results were combined with “malignant” results, the significant asymmetry persists (*p* − value = 0.0009) and agreement between the two methods remains fairly low (16 out of 29 = 55.2%, *p* − value = 0.356). In fact, when aggregated this way, CEUS never reversed a benign CT/MRI diagnosis but often reversed a malignant CT/MRI diagnosis.

**Figure 2:**
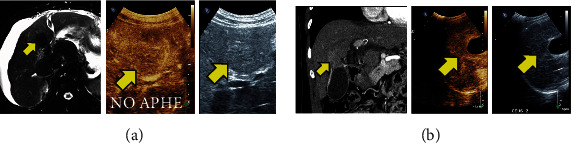
(a) T2-weighted MRI of the liver (left panel) demonstrates a T2 hypointense lesion (arrow) in the central liver. Although a discrete nodule was visualized on grayscale US (right panel), no arterial phase hyperenhancement (APHE) was seen on CEUS (middle panel) consistent with a cirrhotic-associated regenerative nodule (CEUS LI-RADS 3). (b) Coronal contrast-enhanced CT image (left panel) demonstrating a hyperdense region adjacent to the gallbladder (arrow). This region enhances similar to background parenchyma on CEUS (middle panel), and no discrete lesion is visualized on greyscale US (right panel) consistent with a pseudolesion.

The LI-RADS classification system was used as the basis for the evaluation of lesions in the CEUS [7]. CEUS LI-RADS provides a classification system in patients with cirrhosis or with increased risk of cirrhosis which categorizes observations in the liver from LR-1 (definitely benign) through to LR-5 (definitely HCC) according to enhancement patterns and size of the observations (Table 2). Observation is defined as a distinctive area of imaging that differs from the rest of the hepatic parenchyma, which could be lesion or a pseudolesion. CEUS helps confirm the presence of a lesion or pseudolesion (Figure 2) and further categorize it based on the above classification system as summarized in Table 2.

## 4. Discussion

Several imaging modalities are available to diagnose liver lesions without the need for tissue sampling or biopsy. Of these, CEUS offers a relatively inexpensive and safe diagnostic modality in patients with FLL especially when contraindications to conventional cross-sectional imaging exist. CEUS is a dynamic real-time imaging technique that can demonstrate enhancement regardless of the inherent timing or duration of enhancement of the liver lesion. Although CEUS has been around for a few decades, its utilization for noncardiac imaging has only been approved by the FDA in the United States in 2016 [8]. Different phases of contrast enhancement can be identified after injection of contrast. The initial arterial phase begins within 10–20 s and lasts for 35–40 s after the injection. The following portal venous phase, marked by the flow of contrast through the portal system, lasts from injection up to 2 min. During this phase, the overall echogenicity of the liver increases. Since the microspheres are strictly intravascular, there is no interstitial/equilibrium phase leakage, unlike in CT/MRI and that is why lesions wash out more on CEUS than CT or MRI [9–17]. Both enhancement and washout characteristics of lesions on CEUS are useful for the detection of malignant FLLs and enable better hepatological staging of oncology patients.

Imaging characteristics associated with malignancy were avid contrast enhancement within the first minute followed by either quick and early washout within the first minute (Cholangiocarcinoma or metastases imaging pattern) or slow and weak washout (HCC) which is often delayed and begins later than 90 s after injection of the microspheres [15, 18].

**Figure 3:**
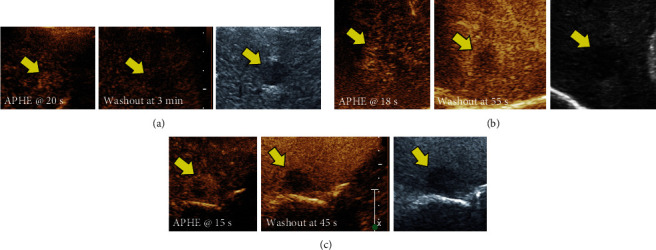
(a) Contrast-enhanced and grayscale US images demonstrate homogenous arterial enhancement (arrow, left panel) of the lesion at 20 s postcontrast injection with weak and slow washout at 3 min (arrow, middle panel) consistent with hepatocellular malignancy. (b) Contrast-enhanced and grayscale US images demonstrating early arterial enhancement at 18 s postcontrast injection (arrow, left panel) with fast and near-complete washout at 55 s (arrow, middle panel) consistent with cholangiocarcinoma (non-HCC malignancy). (c) Contrast enhanced and grayscale US images demonstrating peripheral rim enhancement at 15 s postcontrast injection (arrow, left panel) with fast and near-complete washout at 45 s (arrow, middle panel) consistent with renal cell cancer metastasis (non-HCC malignancy).

The two lesions diagnosed as non-HCC malignancy on CEUS showed a hypoechoic pattern with early arterial phase hyperenhancement within the first 15 s, followed by rapid washout at around 30 s postinjection (Figure 3).

Benign lesions on the other hand show persistent contrast enhancement without washout on the delayed imaging phases.

Hemangiomas are characterized by peripheral, nodular, and discontinuous enhancement in the arterial phase with progressive centripetal fill-in. The late phase shows a complete persistent centripetal fill-in in 40%–50% of cases with persistent hyper or iso-echogenicity [19–23].

**Figure 4:**
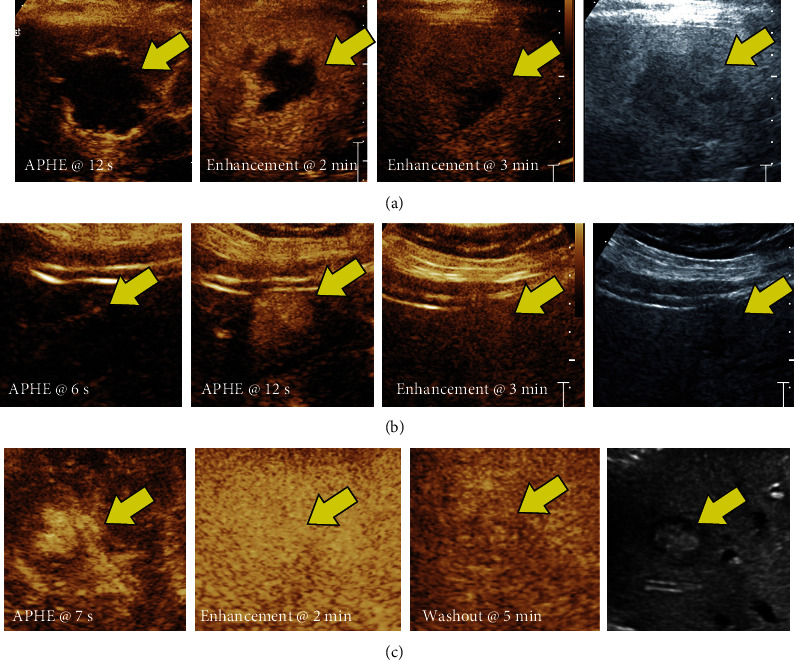
(a) Contrast-enhanced and grayscale US images demonstrating nodular discontinuous peripheral arterial enhancement (arrows) at 12 s postcontrast injection (left panel) with persistent enhancement and fill in at 2−3 min (middle panels) consistent with benign hepatic hemangioma. (b) Contrast-enhanced and grayscale US images demonstrating central to peripheral arterial enhancement (arrows) at 6 s postcontrast injection with growing lesion on arterial phase at 12 s (arrow, second panel) and persistent enhancement at 3 min (arrow, third panel) consistent with follicular nodular hyperplasia (FNH). (c) Contrast-enhanced and grayscale US images demonstrating arterial phase hyperenhancement at 7 s postcontrast injection (arrow, left panel) with centripetal (out-to-in) but non-nodular sustained enhancement at 2 min consistent with hepatic adenoma. Mild washout at 5 min (arrow, third panel) can sometimes be seen with hepatic adenomas which could confuse interpretation.

The pathognomonic feature of focal nodular hyperplasia is a large central fibrous scar in which a feeding artery is located. On CEUS, they appear homogenously hyperechoic in the arterial phase with a characteristic centrifugal fill in which is rather rapid in the early arterial phase [24, 25]. On portal venous and delayed imaging phases, FNHs show persistent contrast enhancement without washout (Figure 4).

**Figure 5:**
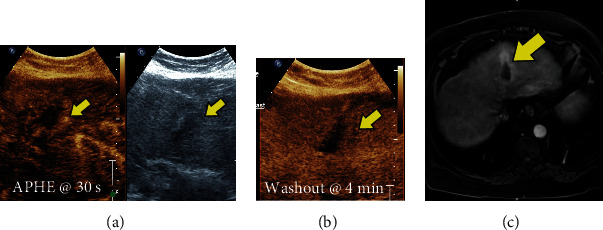
(a) Contrast-enhanced US image (left) and grayscale US image (right) confirming arterial enhancement (arrow) around ablation cavity questioned on (c, arrow) MRI. (b) Delayed washout is also visualized on CEUS at 4 min (arrow) consistent with residual HCC. (c) Axial T1 weighted postcontrast MRI image through the upper abdomen showing contrast enhancement around the ablation cavity (arrow).

Sometimes, CEUS is used to better visualize the arterial enhancement pattern after prior locoregional treatment of a known HCC, and given the entirely intravascular distribution of US microbubbles, avid arterial enhancement in an ablation cavity suggests the presence of residual/recurrent tumor (Figure 5).

Metastases present like FLL or as multiple masses. Previous studies have shown that CEUS has an impressive sensitivity and specificity in the detection of metastasis, ranging from 80% to 95% [26–28]. Most liver metastases are hypovascular or show weak enhancement during the arterial phase which is more pronounced on the periphery of the lesion with a rim-like appearance. On the other hand, hypervascular metastases arise from neuroendocrine tumors (NET); sarcoma, melanoma, renal, thyroid, and breast neoplasms and can show avid solid contrast enhancement. Most hepatic NET metastases show increased arterial enhancement and slower washout and can occasionally wash out after 1 min. Washout in the portal venous phase or the late phase is the most important feature in distinguishing malignant lesions from benign lesions.

In our study, CEUS was able to clearly determine the nature of the lesion in approximately 90% of the patients with indeterminate results on contrast-enhanced cross-sectional imaging. This is very useful in clinical practice as with CEUS positioned as the problem-solving modality, invasive procedures such as tissue sampling can be reduced. Biopsy was rarely recommended during our hepatobiliary tumor board review for these clinical cases. In the 10 cases where biopsy was indicated, CEUS was able to accurately diagnose benign vs malignant lesions which was then confirmed by the biopsy findings in 90% of cases. This highlights the accuracy of CEUS in diagnosing FLLs which results in an improved diagnostic workflow when a FLL is encountered by a clinician and CT/MRI is either indeterminate or the patient cannot get IV contrast for various reasons. This is important in reducing unnecessary biopsies for FLL by using CEUS as a second-line imaging method for indeterminate FLL.

## 5. Conclusion

The earliest mention about the utility of CEUS in the realm of hepatic imaging was in the 2005 edition of the American Association for the Study of Liver Disease (AASLD) guidelines for HCC. However, its use has not come into prominence in the United States until recently, owing to previous conflicting studies and the lack of an affective contrast medium.

Lumason/Sonovue, receiving FDA approval in 2016, expanded the utility of CEUS for evaluating FLLs [8]. In our study, CEUS provided a definitive diagnosis of indeterminate liver lesions in approximately 90% of patients and avoided the need for biopsy in most patients. In those cases where the liver lesions were biopsied, CEUS accurately distinguished malignant versus benign lesions as confirmed by biopsy findings. Our study provides strong evidence supporting the use of CEUS as a reliable and cost-effective diagnostic method for evaluating indeterminate liver lesions. Additionally, it offers the significant benefits of avoiding nephrotoxicity and minimizing the radiation exposure associated with CT or MRI.

## Figures and Tables

**Table 1:** Demographics.

**Variable**	**Sublevel**	**Sample (** **n** ** = 29)**
Age	(Mean ± SD)	54.4 ± 15.4
	(Range)	19 to 77

Gender	Male	11 (38%)
	Female	18 (62%)

Underlying liver disease	Alcohol	4 (14%)
	HBV	1 (3%)
	HCV	4 (14%)
	NAFLD	10 (34%)
	S/P liver transplant	1 (3%)
	None described	9 (31%)

Cirrhosis	No	15 (52%)
	Yes	14 (48%)

BMI	(Mean ± SD)	29.5 ± 6.5
	(Range)	18.9 to 46.6

Prior lesion size (cm)	(Mean ± SD)	2.7 ± 1.4
	(Range)	0.9 to 7.8

**Table 2:** CEUS LIRADS classification (http://www.acr.org/Clinical-Resources/Reporting-and-Data-Systems/LI-RADS/CEUS-LI-RADS).

**CEUS diagnostic table**				
**Arterial phase Hyperenhacement (APHE)**	**No APHE**		**APHE (not rim** ^a^ **, not peripheral discontinuous globular** ^b^ **)**	
Nodule size (mm)	< 20	≥ 20	< 10	≥ 10
No washout of any type	CEUS LR-3	CEUS LR-3	CEUS LR-3	CEUS LR-4
Late and mild washout	CEUS LR-3	CEUS LR-4	CEUSLR-4	CEUS LR-5

*Note:* CEUS LR-M criteria—any of the following: rim APHE OR, early (< 60 s) washout OR, and marked washout. LI-RADS 1 = definitely benign; LI-RADS 2 = probably benign; LI-RADS 3 = intermediate probability for hepatocellular carcinoma; LI-RADS 4 = probably hepatocellular carcinoma; LI-RADS 5 = definitely hepatocellular carcinoma; LI-RADS M = probably or definitely malignant but not HCC specific; LI-RADS TR = equivocal, treated, equivocally viable; LI-RADS TR = nonviable, treated, probably or definitely not viable; LI-RADS TR = viable, treated, probably or definitely viable.

Abbreviations: CT, computed tomography; HCC, hepatocellular carcinoma; HCV, hepatitis C virus; LIRADS, liver imaging reporting and data system; NAFLD, nonalcoholic fatty liver disease; MRI, magnetic resonance imaging; NASH, nonalcoholic steatohepatitis.

^a^Rim APHE indicates LR-M.

^b^Peripheral discontinuous globular indicates hemangioma (LR-1).

## Data Availability

The manuscript data used to support the findings of this study are available from the corresponding author upon request.
